# Genomic Analysis of *Xanthomonas translucens* Pathogenic on Wheat and Barley Reveals Cross-Kingdom Gene Transfer Events and Diverse Protein Delivery Systems

**DOI:** 10.1371/journal.pone.0084995

**Published:** 2014-01-09

**Authors:** Donald M. Gardiner, Narayana M. Upadhyaya, Jiri Stiller, Jeff G. Ellis, Peter N. Dodds, Kemal Kazan, John M. Manners

**Affiliations:** 1 Commonwealth Scientific and Industrial Research Organisation, Plant Industry, St Lucia, Brisbane, Queensland, Australia; 2 Commonwealth Scientific and Industrial Research Organisation, Plant Industry, Black Mountain, Canberra, Australian Capital Territory, Australia; Cankiri Karatekin University, Turkey

## Abstract

In comparison to dicot-infecting bacteria, only limited numbers of genome sequences are available for monocot-infecting and in particular cereal-infecting bacteria. Herein we report the characterisation and genome sequence of *Xanthomonas translucens* isolate DAR61454 pathogenic on wheat and barley. Based on phylogenetic analysis of the ATP synthase beta subunit (*atpD*) gene, DAR61454 is most closely related to other *X. translucens* strains and the sugarcane- and banana- infecting *Xanthomonas* strains, but shares a type III secretion system (T3SS) with *X. translucens* pv. *graminis* and more distantly related xanthomonads. Assays with an adenylate cyclase reporter protein demonstrate that DAR61454's T3SS is functional in delivering proteins to wheat cells. *X. translucens* DAR61454 also encodes two type VI secretion systems with one most closely related to those found in some strains of the rice infecting strain *X. oryzae* pv. *oryzae* but not other xanthomonads. Comparative analysis of 18 different *Xanthomonas* isolates revealed 84 proteins unique to cereal (i.e. rice) infecting isolates and the wheat/barley infecting DAR61454. Genes encoding 60 of these proteins are found in gene clusters in the *X. translucens* DAR61454 genome, suggesting cereal-specific pathogenicity islands. However, none of the cereal pathogen specific proteins were homologous to known *Xanthomonas* spp. effectors. Comparative analysis outside of the bacterial kingdom revealed a nucleoside triphosphate pyrophosphohydrolase encoding gene in DAR61454 also present in other bacteria as well as a number of pathogenic *Fusarium* species, suggesting that this gene may have been transmitted horizontally from bacteria to the *Fusarium* lineage of pathogenic fungi. This example further highlights the importance of horizontal gene acquisition from bacteria in the evolution of fungi.

## Introduction

Bacterial pathogens are of serious economic importance in many crop plants. In monocotyledonous plant hosts, *Xanthomonas* pathogens cause important diseases such as rice blight and sugarcane leaf scald caused by *X. oryzae* pv. *oryzae* and *X. albilineans*, respectively. Although bacterial diseases of the important cereals wheat and barley are economically less important than fungal diseases, recent genome analyses of fungal pathogens have indicated bacteria, and particularly plant-associated bacteria, are potential sources of new virulence genes contributing to the evolution of fungal virulence [1]. For example, a comparative analysis of the genome of the cereal crown rot pathogen *Fusarium pseudograminearum* identified 17 genes exclusively found in cereal pathogens and possibly acquired from bacteria by horizontal transfer. One of these genes encoding an amidohydroylase was shown to be required for pathogen virulence on both wheat and barley [2]. Similarly, Klosterman *et al.* [3] showed that a glucosyltransferase important for virulence in the wilt causing *Verticillium* fungi likely originated from bacteria. So far, horizontally acquired genes with highly uneven distributions across fungal species but close protein matches from bacterial species have primarily been discovered through the analysis of sequenced fungal genomes. Similarly, examining the genomes of phytopathogenic bacteria for genes that appear preferentially in other pathogens specialised to particular hosts could reveal new insights into the evolution of pathogenicity and/or virulence. Application of this approach to bacteria pathogenic on cereals requires the availability of sequenced genomes from these pathogens and through this and other efforts we have sought to expand the available genomes [4].

Bacterial pathogens and non-pathogens are increasingly utilised as a vehicle to deliver heterologous proteins into plant cells to dissect functions of pathogen proteins with potential roles in virulence [5]. So far, such protein delivery systems have primarily been exploited in dicotyledonous model hosts such as *Nicotiana benthamiana* and *Arabidopsis thaliana* [6]. Development of similar systems in monocots using appropriate bacterial pathogens would also be highly desirable to analyse effector function. Previous systems utilizing non-pathogenic strains of *Pseudomonas* have been described [7] and we recently developed a similar system with improved phenotypic penetrance in cereal in searching for resistance/avirulence gene interactions [8]. The delivery of effectors using pathogenic strains that cause cell death will also be useful to identify effectors that contribute to virulence functions such as suppression of cell death.

We report herein the genome sequence of a *X. translucens* isolate pathogenic on wheat and barley. We have used the *X. translucens* genome sequence to search for bacterial genes that may have been acquired by fungal plant pathogens. We have also demonstrated the ability of this pathogen to deliver heterologous proteins into wheat cells. An improved knowledge of the genome and effector composition produced by plant pathogenic bacteria may provide a useful tool for functional genomics research on effector proteins acting on cereal hosts.

## Results and Discussion

### *Xanthomonas translucens* DAR61454 is a wheat and barley pathogen

**Figure 1:**
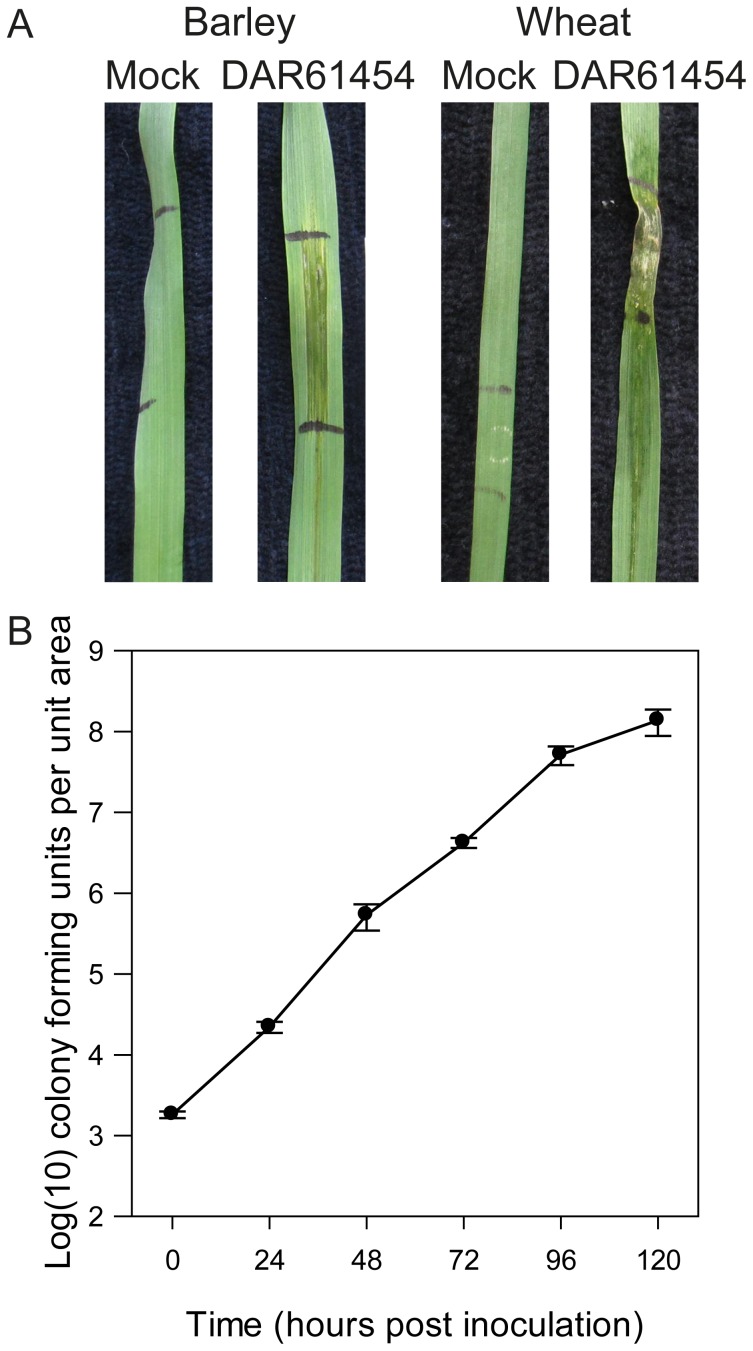
Virulence of *Xanthomonas translucens* DAR61454 towards wheat and barley. (A) Exemplar photographs of virulence of *X. translucens* DAR61454 on wheat and barley (six days post inoculation). Black marks on the leaves highlight the extremities of the bacterial infiltration (B) Bacterial growth over time during infection of barley. No rifampicin resistant bacteria were recovered from mock inoculated plants carried out at the same time. Each time-point is average of four biological replicates with error bars representing the standard error of the mean. One unit area is two leaf disc punches.

Despite the demonstrated importance of bacterial species as potential donors of virulence genes in fungal cereal pathogens [reviewed in 1], currently limited genome sequence information is available for cereal infecting bacteria and this makes searching for new such genes difficult. In this study, we first aimed to identify an Australian bacterial strain capable of infecting cereal hosts, given that the importation of plant pathogens into Australia is restricted by quarantine laws. *Xanthomonas* strain DAR61454 identified as *X. translucens* was isolated from wheat showing symptoms of black chaff disease at a research station in Tamworth, New South Wales, Australia in 1988. To confirm the pathogenicity of this isolate on cereals, we generated a rifampicin resistant derivative of DAR61454 and infiltrated this strain into wheat and barley leaves. Lesions in the infiltrated zone were observed after inoculations in both species (Figure 1A). These lesions expanded over time and became water-soaked and necrotic, consistent with descriptions of *X. translucens* pathogenicity on wheat [9]. In addition, bacterial concentrations increased exponentially over time in infiltrated barley leaves, consistent with a virulent interaction on this species (Figure 1B). The pathogenicity of DAR61454 was also tested in over 100 wheat accessions which all produced water-soaked lesions following inoculation (data not shown). Together, these experiments indicate that DAR61454 is highly pathogenic on both wheat and barley.

### Genome assembly and annotation

The DAR61454 genome was sequenced using 100 bp paired end Illumina reads. The total assembly size was 4.5 Mb assembled in 217 scaffolds (split into 404 contigs for annotation). The average coverage depth was 1585 fold. The N75 (number of scaffolds for which 75% of the genome is contained) was 68. The genome was annotated using the NCBI Prokaryote Genome Automatic Annotation Pipeline (PGAAP). The locus tag prefix assigned to this isolate is A989. The genome was predicted to encode 3847 proteins. The sequence data from this whole genome shotgun project has been deposited to DDBJ/EMBL/GenBank under the accession AMXY00000000. The version described in this paper is the first version, AMXY01000000. The raw sequence reads have been deposited into the NCBI sequence read archive under accession SRS502548.

### Comparative genome analyses within *Xanthomonas* spp

**Figure 2:**
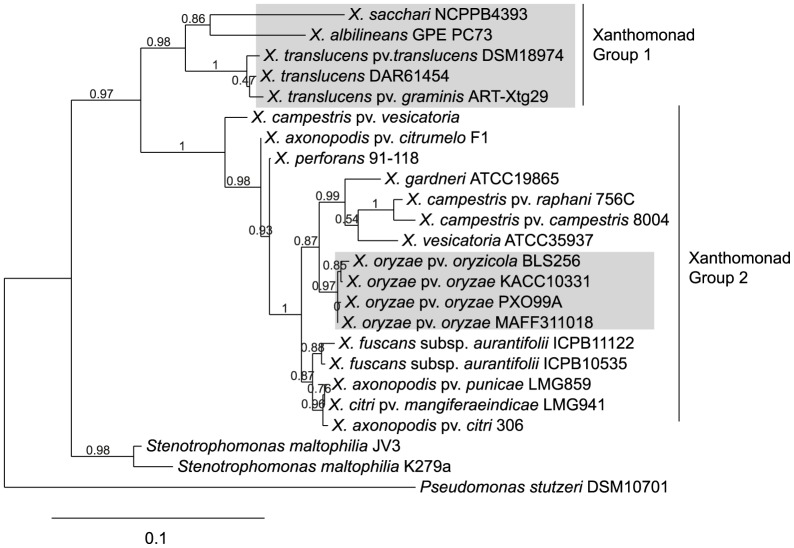
Placement of *Xanthomonas translucens* isolate DAR61454 in the *Xanthomonas* genus using phylogenetic analysis of the *atpD* gene nucleotide sequence. Phylogenetic analysis was carried out at www.phylogeny.fr as detailed in the methods. Numbers of branches represent branch support based on approximate likelihood ratio testing. Monocot infecting or associated xanthomonads are shaded in grey. All other xanthomonads are associated with dicot species.

We used the ATP synthase beta subunit encoding gene (*atpD*) sequence to analyse phylogenetic relationships between DAR61454 and other xanthomonads. This gene has previously been used to infer phylogenetic relationships within xanthomonads [10]. As expected, the best nucleotide matches of DAR61454 *atpD* were from other *X. translucens* strains with pathovar specifications of either *translucens* or *graminis*. These related sequences have been very recently deposited into GenBank; however, a detailed description of one of these *X. translucens* genomes was only published whilst this manuscript was under review [11]. The *Xanthomonas* genus consists of two distinct phylogenetic groups, with most of the extant genomes from isolates in Group 2 [10]. Genome sequences of Group 1 xanthomonads are currently limited to three recently sequenced banana-associated isolates [10], the sugarcane pathogen *X. albilineans* [12] and the new *X. translucens* submissions to GenBank. Phylogenetic analysis using *atpD* suggested DAR61454 belongs to the Group 1 strains of the genus (Figure 2). The *gyrB* gene, which encodes the B subunit of the DNA gyrase, provides additional resolution for intraspecific identification of xanthomonads [13]. Therefore, additional phylogenetic analysis was carried out using *gyrB* and, as expected, DAR61454 grouped with other *X. translucens* pv. *translucens* strains. However, the branch support for separating the *X. translucens* pv. *translucens* group from the single strain of *X. translucens* pv. *graminis* was weak (Figure S1). The host infection phenotypes together with phylogenetic analyses of these two genes strongly support the classification of DAR61454 as *X. translucens* pv. *translucens*.

### Orthologous proteins in *Xanthomonas* spp

To further analyse the DAR61454 genome, 18 sequenced xanthomonad genomes including DAR61454 were compared using orthoMCL cluster analysis [14]. The genome sequences used in this analysis were obtained from www.xanthomonas.org, which has gene annotations associated with the GenBank entries (Table 1). OrthoMCL groups proteins into putative orthologous groups based on sequence similarity. A total of 6590 different groups of proteins were identified in the 18 genomes analysed. Of these, 912 groups of proteins were unique to monocot infecting (sugarcane, rice and wheat), while 727 were specific to cereal infecting (rice and wheat) isolates. The accessions for these 727 cereal specific groups of proteins have been included in File S1. These included 84 proteins (in 74 groups) from DAR61454. Sixty of these 84 proteins were present in clusters of two or more genes in the DAR61454 genome, suggestive of gene transfers of contiguous segments or selective retention of physically co-located genes between cereal infecting xanthomonads, despite the relatively distant overall evolutionary relationship between DAR61454 and the rice infecting *Xanthomonas* spp. Two large gene clusters, one encoding a type VI secretion system (T6SS, see below) and the other one encoding a number of cytochrome P450 monooxygenases and two polyprenyl transferases (A989_04693-A989_04733), were identified in protein groups specific to cereal infecting isolates. The latter cluster encodes enzymes with closest matches in rhizobial species with the predicted gene functions, suggestive of a secondary metabolite synthetic pathway. One of the most important features of the virulence arsenal of the sugarcane pathogen *X. albilineans* is the production of the polyketide phytotoxin albicidin [15], but DAR61454 does not encode the genes required for the production of this toxin.

**Table 1:** *Xanthomonas* spp. genomes used in comparative analysis.

Isolate abbreviation	Species	Host(s)	GenBank Accession(s)
**A989**	*Xanthomonas translucens*. DAR61454	wheat and barley	AMXY01000000
**PXO**	*Xanthomonas oryzae* pv. *oryzae* PXO99A	rice	CP000967
**XAC**	*Xanthomonas axonopodis* pv. *citri* str. 306	citrus	AE008923, AE008924, AE008925
**XACM**	*Xanthomonas axonopodis pv. citrumelo* F1	citrus	CP002914
**XALC**	*Xanthomonas albilineans* GPE PC73	sugarcane	FP565176
**XAPC**	*Xanthomonas axonopodis pv. punicae* str. LMG 859	pomegranate	CAGJ01000001
**XAUB**	*Xanthomonas fuscans* subsp. *aurantifolii* str. ICPB 11122	citrus	ACPX00000000
**XAUC**	*Xanthomonas fuscans* subsp. *aurantifolii* str. ICPB 10535	citrus	ACPY00000000
**XCXX**	*Xanthomonas campestris pv. campestris* str. 8004	cauliflower	CP000050
**XCR**	*Xanthomonas campestris* pv. *raphani* 756C	*Brassica oleracea* var *capitata*	CP002789
**XCV**	*Xanthomonas campestris* pv. *vesicatoria* str. 85–10	pepper	AM039948, AM039949, AM039950, AM039951, AM039952
**XGA**	*Xanthomonas gardneri* ATCC 19865	tomato and pepper	AEQX01000000
**XMIN**	*Xanthomonas citri* pv. *mangiferaeindicae* LMG 941	mango	CAHO01000001
**XOC**	*Xanthomonas oryzae* pv. *oryzicola* BLS256	rice	CP003057
**XOOB**	*Xanthomonas oryzae* pv. *oryzae* MAFF 311018	rice	AP008229
**XOO**	*Xanthomonas oryzae* pv. *oryzae* KACC10331	rice	AE013598
**XPE**	*Xanthomonas perforans* 91–118	tomato	AEQW01000000
**XVE**	*Xanthomonas vesicatoria* ATCC 35937	tomato and pepper	AEQV00000000

### Secretion systems

Gram negative bacteria can have up to six different types of specialized secretion systems to deal with the unique dual membrane system found in these organisms [16]. Direct roles in virulence towards plants have been described for the T1SS, T2SS, T3SS, T4SS and T6SS [17]–[20]. Using the *X. oryzae* pv. *oryzae* T1SS protein sequences (PXO_04478 (RaxA), PXO_04477 (RaxB) and PXO_02621 (RaxC)) as a query, we identified orthologous sequences in the DAR61454 genome (A989_01015, A989_01020 and A989_15402). The T2SS is encoded by 12 or more genes [21]. *X. axonopodis* pv. c*itri, X. campestris* pv. *campestris and X. citri* contain two T2SS [22], [23] whereas *X. oryzae* pv. *oryzae* encodes one [24]. DAR61454 contains one gene cluster for this system with a typical organization with xpsE through xpsN encoded in one cluster (*A989_04551*-*A989_04506*) with xpsD (*A989_017673*) and xpsO (*A989_03190*) encoded at other genomic locations. Apart from the separate location of *xpsD*, the cluster is arranged the same as *X. campestris* pv. *campestris* [21].

There does not appear to be a T4SS in DAR61454. *X. axonopodis* pv. c*itri* encodes two T4SSs, one each on the chromosome and plasmid [25] and *X. campestris* pv. *campestris* encodes one T4SS cluster [22]. BLASTp searches using these sequences against DAR61454 failed to identify orthologous proteins, with the exception of a single virD4 homologue encoded by A989_18933.

**Figure 3:**
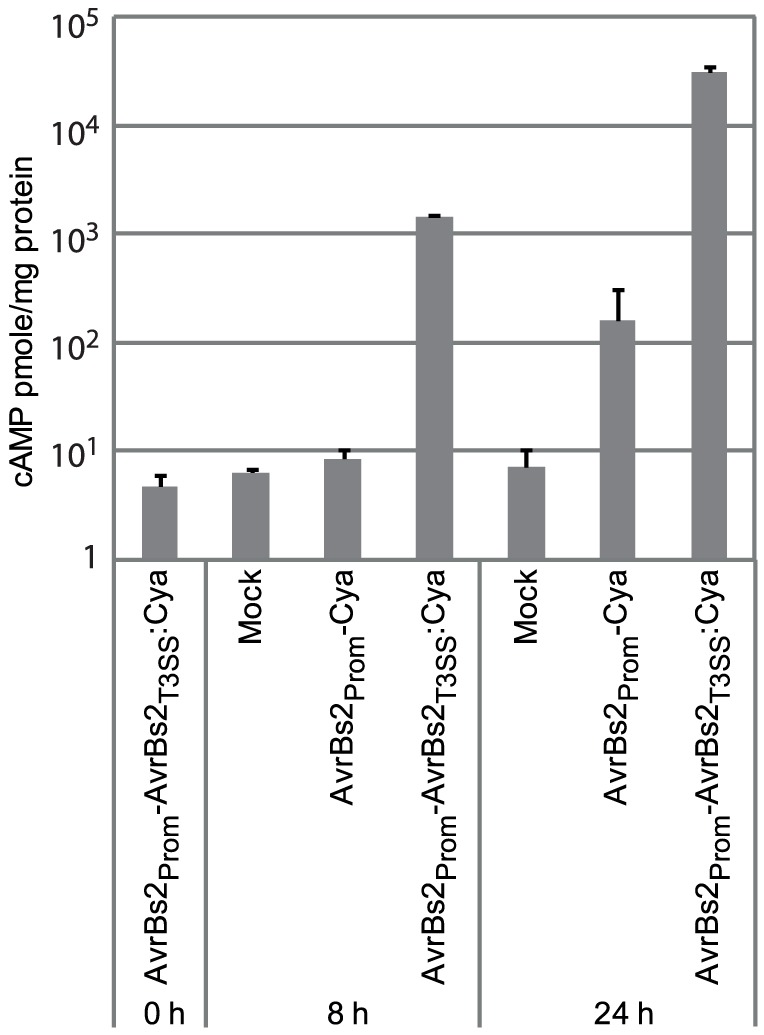
Use of *Xanthomonas translucens* DAR61454 to deliver a heterologous reporter protein into wheat cells via the Type III secretion system (T3SS). The uniquely eukaryote-functioning calmodulin dependent adenylate cyclase (Cya) reporter was translationally fused to AvrBs2 N-terminus leader in a bacterial expression vector (AvrBs2_Prom_-AvrBs2_T3SS_∶Cya). cAMP detection is indicative of delivery of the Cya protein into host cells which was measured by an enzyme immunoassay. The mock treatment used MgCl_2_ without bacteria. A construct lacking the AvrBs2 T3SS signal but with the *AvrBs2* promoter and Cya sequence (AvrBs2_Prom_-Cya) was used as a control. Some background Cya activity could be observed at the later time point with this construct. The data is average of three biological replicates and error bars represent the standard error of the mean.

Our analyses revealed that, in contrast to *X. albilineans*, which contains a *Salmonella* SPI-1 type III secretion system (T3SS), and the banana-associated strains that lack a T3SS [10], DAR61454 encodes a Hrp-type T3SS cluster (A989_10802-A989_10942) that most closely resembles that found in the recently sequenced *X. translucens* pv. *graminis* genome and those of Group 2 xanthomonads [11]. The organization of the cluster is almost identical to that described *X. translucens* pv. *graminis* [11] with the exception of an additional hypothetical protein (A989_10862) encoded between the genes for *hpaP* and *hrcV*. Given the central importance of T3 effectors in bacterial virulence in both plants and animals, mutation of the T3SS in xanthomonads typically results in a loss of virulence and this was recently demonstrated for *X. translucens* pv. *graminis* [8]; so it can be expected the DAR61454 T3SS is also important for virulence. The identification of the conserved Hrp-T3SS in the DAR61454 genome suggested this strain could be used as a vehicle to deliver heterologous proteins from other pathogens of cereals to understand their roles in virulence. To demonstrate the potential utility of this strain, a *X. campestris* AvrBs2:calmodulin-dependent adenylate cyclase (Cya) fusion reporter system was used [26], [27]. This reporter system relies on the presence of calmodulin exclusively in eukaryotic cells, meaning that the Cya protein is inactive unless present inside a eukaryotic cell. Thus, the accumulation of cyclic adenosine monophosphate (cAMP) above control levels is dependent on delivery of the calmodulin-dependent-Cya to the host cells. Using this system, we observed significant cAMP accumulation, normalized to total protein content, in wheat leaves 8 and 24 hours after inoculation with *X. translucens* DAR61454 carrying an AvrBs2_T3SS_∶Cya expressing plasmid (expression driven by AvrBs2 promoter) in which Cya was translationally fused to the first 100 amino acids of AvrBs2 including its T3SS signal (Figure 3). As a control, a vector containing the same promoter driving Cya expression but lacking the AvrBs2 T3SS signal sequence was used to demonstrate the dependence of the observed Cya activity on the AvrBs2 T3SS. Only low levels of cAMP were detected in leaves infiltrated with this construct, approximately 100-fold lower than that observed with AvrBs2_T3SS_∶Cya construct at 24 hours (Figure 3). The levels of cAMP observed using DAR61454 to deliver Cya are similar to those observed using this type of reporter system in other bacteria-plant interactions [8], [28], [29]. This result clearly demonstrates the ability of DAR61454 to deliver the reporter protein into wheat cells.

**Figure 4:**
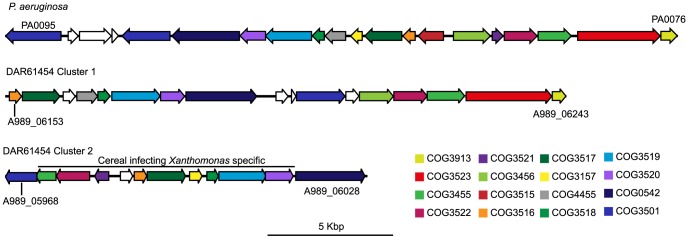
Type VI secretion system (T6SS) gene clusters in DAR61454. The upper cluster shows the prototypical T6SS from *Pseudomonas aeruginosa*. DAR61454 Cluster 1 represents the T6SS cluster found in most *Xanthomonas spp*. genomes and Cluster 2 appears to be specific to DAR61454 and rice infecting isolates. Orthologous genes are color coded according to their cluster of orthologous groups of proteins (COG) classification in the annotated genomes. Color coding is an approximate of that used by Boyer et al. [30]. Gene numbers at the extremities of the clusters are shown but other numbers are omitted for ease of viewing. In DAR61454 Cluster 1 genes A989_06178 and A989_06183 (COG3519) and Cluster2 genes A989_05968 and A989_05973 (COG3501) were on either side of scaffold gaps and have been fused in this image as they appear to be parts of the same genes.

DAR61454 also encodes two type VI secretion system (T6SS) gene clusters which both contain nearly all the conserved elements of these clusters (Figure 4) as defined by Boyer et al. [30]. The T6SS was originally identified as a secretion system in the mammalian infecting pathogens *Pseudomonas aeruginosa* and *Vibrio cholerae* where it is important for virulence [31], [32]. The T6SS is also used for the secretion of proteins that alter the ability of rhizobia to form functional nodules in a host specific manner [33]. T6SSs are typically encoded by 15–20 genes with 13 of these present at most T6SS loci in different species [30]. One T6SS cluster (A989_06153-A989_06243; Cluster 1) in DAR61454 appears to be found in most Xanthomonads. In contrast, 10 of the 12 genes in the other cluster (A989_05968-A989_06028; Cluster 2) (Figure 4) were identified as cereal specific using the OrthoMCL clustering analysis. Outside of these matches, the next best hits for each of the proteins of the DAR61454 T6SS are to those from a *Stenotrophomonas* sp., which is a bacterial genus often found in soil and rhizophere samples [34]. These secretion system clusters identified in DAR61454 are likely to be involved in the plant-associated lifestyle of this isolate. Exactly how two separate T6SS that presumably recognise and secrete similar proteins can function in a single bacterium remains obscure.

### Ef2fector homologues

BLASTp analysis of the DAR61454 genome revealed the presence of type III effector homologues from 22 different families of *Xanthomonas* effectors, as classified at www.xanthomonas.org. These DAR61454 effector homologues are listed in Table 2. None of these known effectors are exclusively present in the cereal pathogens. However, it is likely that different effector combinations and/or specific allelic variants of these effectors together with other virulence factors would determine host specificity, as has been suggested for both *X. axonopodis* [35] and *Pseudomonas syringae* isolates [36], rather than individual effectors providing the ability to invade a particular host. For five classes of effectors (XopF1, F2, L, X and AE), multiple homologues are present in the DAR61454 genome. DAR61454 is also likely to encode TAL (transcription activator-like) type effectors based on BLASTp analysis. However, DAR61454 genomic regions containing matches to these genes were found on six high coverage contigs which appear to be poorly assembled due to the inherent repetitive nature of TAL-encoding genes [37]. Extraction of the reads from these contigs and assembly against TAL effector coding sequences from *X. oryzae* pv. *oryzae* as a reference suggested that there was at least 15 Kbp of sequence in DAR61454 at a coverage equivalent to 1585-fold in these sequence reads. With *Xanthomonas* TAL effector encoding sequences typically in the range of 3–5 Kbp, this could indicate that there are at least 3–5 TAL effectors in DAR61454. However, longer sequence reads are needed to resolve these.

**Table 2:** Known effector homologues in *Xanthomonas translucens* DAR61454.

A989 protein	Xop Nomenclature	% identity	E-value
**A989_10180**	AvrBs2	58.82	0
**A989_12395**	XopB	53.3	6e^−169^
**A989_02940**	XopC2	63.56	5e^−161^
**A989_10570**	XopF1	57.37	1e^−169^
**A989_10802**	XopF1	33.51	8e^−63^
**A989_10570**	XopF2	76.83	0
**A989_10802**	XopF2	34.98	5e^−72^
**A989_02970**	XopJ5	88.94	2e^−116^
**A989_13784**	XopK	63.75	0
**A989_06893**	XopL	40.06	5e^−113^
**A989_16433**	XopL	30.63	2e^−31^
**A989_07258**	XopN	58.66	0
**A989_02550**	XopAK	63.1	1e^−67^
**A989_08274**	XopP	64.98	0
**A989_00475**	XopP	39.85	1e^−92^
**A989_14199**	XopQ	47.89	9e^−95^
**A989_09773**	XopR	44.68	7e^−29^
**A989_17343**	XopV	52.34	1e^−64^
**A989_02945**	XopX	60.93	0
**A989_00070**	XopX	57.86	2e^−178^
**A989_00075**	XopX	53.49	5e^−178^
**A989_04281**	XopZ1	57.65	0
**A989_03235**	XopAA	80.2	0
**A989_04481**	XopAD	67.27	0
**A989_06893**	XopAE	32.41	1e^−26^
**A989_16433**	XopAE	31.48	7e^−10^
**A989_08851**	XopAF	33.1	5e^−19^
**A989_07073**	XopAH	32.29	2e^−33^

Proteins in this strain have the prefix A989_ supplied by GenBank followed by a unique number.

### Evidence for a bacterial to fungal gene transfer

**Figure 5:**
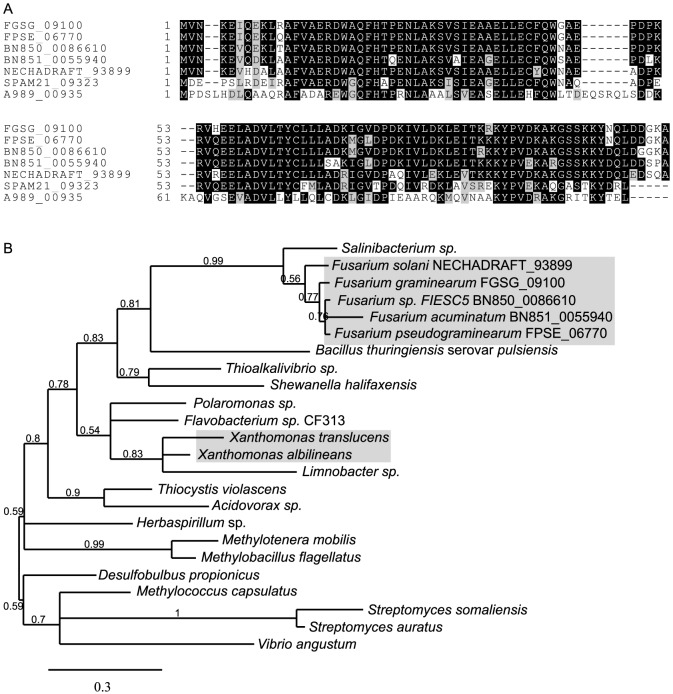
Sequence similarities between nucleoside triphosphate pyrophosphohydrolases (NTPHs) in bacteria and fungi. (A) Multiple sequence alignment of the only three fungal NTPHs in GenBank with those of two bacteria. FGSG_09100, FPSE_06770, BN850_0056610, BN851_0055940 and NECHADRAFT_93899 are sequences from *Fusarium graminearum*, *F. pseudograminearum, F. sp.* FIESC5, *F. acuminatum* and *F. solani* f. sp. *pisi*, respectively SPAM21_09323 and A989_00935 are sequences from *Salinibacterium* sp. strain PAMC21357 and *Xanthomonas translucens* DAR61454 respectively. (B) Phylogenetic analysis of the NTPH protein sequence found in *Fusarium* spp. and bacteria. The *Fusarium* and *Xanthomonas* clades are shaded with all other sequences from bacteria. Analysis was conducted at phylogeny.fr as detailed in the methods. Numbers on each branch indicate support for that branching based on approximate likelihood ratio tests.

Lateral or horizontal gene transfer is one possible route by which fungal species can acquire new functions to facilitate niche colonisation or gain a competitive advantage. In our recent analysis of the genome of the wheat pathogen *F. pseudograminearum*, a number of bacterial to fungal gene transfers were identified with strong phylogenetic support [2]. These analyses prompted the sequencing of bacterial pathogens of wheat and barley, such as DAR61454 described here, as possible sources of such horizontally-derived virulence genes. BLASTp analysis of the grass pathogen-specific set of proteins against NCBI's non-redundant (nr) protein database restricted to fungal entries identified a protein encoding a nucleoside triphosphate pyrophosphohydrolase (NTPH) shared between DAR61454 (A989_00935) and the sugarcane pathogen *X. albilineans*. Interestingly, this gene also had homologues in plant infecting *Fusarium* species (*F. graminearum*, *F. pseudograminearum, F. solani* f. sp. *pisi*, *F*. *acuminatum* and species five of the *F. incarnatum-equiseti* species complex (*F.* sp. FIESC5)). These matches to A989_00935 from these five related fungi were the only ones found in the fungal databases at NCBI and whilst only 45% identical, the matches to fungal sequences were throughout the entire length of the proteins at an e-value of 2×10^−25^. Furthermore, when the *Fusarium* sequences were used as a query in BLASTp searches, the best hit was to a *Salinibacterium* sp. with 67% sequence identity and e-value of 2×10^−48^. The similarity between each of these sequences is shown in a multiple alignment in Figure 5A. Phylogenetic analysis along with the extremely limited distribution of this sequence in fungi supports the view that these three *Fusarium* spp. acquired this gene from bacteria, but xanthomonads do not appear to be the likely donor genus (Figure 5B). Inspection of the genomic regions of these homologues showed synteny between the A989_00935 homologues and flanking genes in *F. graminearum* and *F. pseudograminearum* [2], [38] is conserved. Given these lineages separated ∼1.7 million years ago, the transfer into these species most likely predates this divergence. Synteny is not maintained between the *F. pseudograminearum*/*F. graminearum* region and *F. solani* f. sp. *pisi*, *F. acuminatum* or *F.* sp. FIESC5 regions [39], [40]. NTPHs of this class are involved in the regulation of a cell death pathway encoded by a toxin-antitoxin system in bacteria and form part of a polycistronic RNA with the toxin and antitoxin proteins [41]. However, based on the absence of the other components of this toxin-antioxin system in the genomic region of A989_00935 and the homologue from *Salinibacterium* sp., these bacterial and fungal homologues may relate to the NTPH enzymatic activity but not in the regulation of toxin-antitoxin systems. Thus a role for NTPHs in bacterial or fungal pathogenesis on plants currently remains obscure.

In summary, the DAR61454 genome sequenced and initially characterised in this work will be an important resource for comparative analyses of pathogenicity in bacterial and fungal cereal pathogens. In addition the demonstrated ability of DAR61454 as a tool to deliver proteins to the cytoplasm of cereal cells using the type III secretion system can be exploited for molecular pathology studies in a number of plant pathogen interactions. The T6SS found in DAR61454 may also be useful for the delivery of proteins extracellularly, as at least in some bacteria, substrates of this secretion system can be found in culture filtrates [31], although the signature sequences involved in type VI secretion systems are yet to be fully characterised. Finally, the identification of yet another phylogenetically supported horizontal gene acquisition by fungal plant pathogens adds to the growing body of evidence that the acquisition of new genes from bacteria has been a useful evolutionary strategy in fungi. Further sampling of plant associated bacteria via genome sequencing and metagenomics is clearly warranted to fully understand the pervasiveness of this phenomenon in the evolution of virulence in fungal pathogens.

## Experimental Procedures

*Xanthomonas* strain DAR61454, identified as *X. translucens*, was isolated in the Tamworth region of New South Wales, Australia in March 1988 from wheat and is available from the Australian Collection of Plant Pathogenic Bacteria in the New South Wales Department of Primary Industries. *X. translucens* DAR61454 was cultured using King B media at 28°C. A rifampicin resistant strain of *X. translucens* DAR61454 was generated by plating cells from a liquid culture on a King B plate containing a gradient of rifampicin [42] and a single colony picked from the region containing high levels of the antibiotic.

### Pathogenicity of *X. translucens* DAR61454

Pathogenicity of *X. translucens* DAR61454 was established using two different assays. For visual symptom assessment, a three day culture was diluted to an optical density at 600 nm of 0.05 in 10 mM MgCl_2_ and approximately 200 µL of this suspension was infiltrated using a 1 mL needleless syringe in to the adaxial side of the second leaf of 10 day old wheat or barley seedlings until the infiltrated liquid started escaping out of stomata. Typically the leaf zone that received inoculum was a 1-2 centimetres either side of the infiltration point. Plants were grown in a glasshouse maintained at 24/16°C and 60/90% relative humidity day/night without any supplementary lighting. For the assessment of bacterial growth over time, a 100-fold dilution of an OD_600_ = 0.05 suspension of culture of a rifampicin resistant derivative of DAR61454 was made in 10 mM MgCl_2_ and infiltrated in the same manner as above. The region infiltrated was marked with a permanent pen. Two 7 mm-diameter leaf disks were harvested, one above and one below the infiltration site but both within the infiltrated zones, from each leaf and transferred to a 2 mL eppendorf tube with a single 3 mm ball bearing. The material was ground in a Retsch mill for 30 s at 30 Hz. 400 µL of 10 mM MgCl_2_ was added to the tube before it was briefly vortexed. A dilution series from each tube was made across six orders of magnitude and 10 µL of these were plated onto King B media containing rifampicin at 50 mg L^−1^. Bacteria were allowed to grow for two nights at 28°C prior to counting colonies. Each time point consisted of four biological replicates.

### Genome sequencing

DNA for genome sequencing was prepared from bacteria grown in liquid culture using a QIAgen Blood and Tissue kit according to the manufacturer's instructions. Illumina library preparation and sequencing was performed by the Australian Genome Research Facility, Melbourne, Australia using one fifth of a HiSeq2000 lane for the sequence generation. Following quality filtering (low quality threshold of 0.05, maximum number of ambiguities set at 2, the removal of 2 terminal nucleotides and a minimum length of 50 bases) of paired end Illumina reads, the genome was assembled from 7.4×10^7^ reads using CLC Genomics Workbench version 5.1 with the scaffolding option selected (minimum contig size 200, minimum and maximum paired end distances set at 316 and 666 bp, respectively. Low coverage contigs (<500-fold with all but four of these being below 100 bp) were removed from the final assembly. As the assembly and scaffolding occurred in one operation in the CLC Genomics Workbench environment, to allow submission to the NCBI Prokaryotic Genome Annotation Pipeline (PGAAP), the 217 scaffolds were split into 404 contigs using custom Perl scripts developed in house (available upon request). Contig relationships were maintained in the GenBank submission by inclusion of a Golden Path (agp) file. No additional curation of the PGAAP annotation was undertaken.

### Phylogenetic analysis

The *atpD* gene was used to infer the taxonomic position of *X. translucens* DAR61454 based on the analysis performed by Studholme *et al*. [10]. To provide resolution at the pathovar level, the *gyrB* gene sequence was used including sequences from the strains described by Parkinson et al. (NCBI population set accession 151936706) [13]. Analysis was performed on nucleotide sequences for these genes at www.phylogeny.fr [43] using a MUSCLE alignment [44], GBLOCKS curation of the nucleotide alignment [45], the PhyML phylogeny package [46] and the HKY85 nucleotide substitution model with approximate likelihood-ratio test for branch support [47]. Tree rendering was performed with TreeDyn.

Phylogenetic analysis of the nucleoside triphosphate pyrophosphohydrolase found in *Fusarium* spp. and bacteria was performed as described above but on protein sequences manually selected from BLASTp queries at NCBI to represent taxonomically diverse hits to the sequence. The Whelan and Goldman (WAG) evolutionary model for protein sequences was used. Tree rendering was performed with TreeDyn.

### Determining protein orthologous relationships

OrthoMCL [14] was implemented on a desktop computer running Ubuntu 12.04 operating system with 16 Gb of RAM. Protein sequences from each of the isolates were extracted from the GenBank entry using custom Perl scripts. Locus tags identifiers were modified for those GenBank entries that did not conform to the three or four character prefixes required by OrthoMCL using custom Perl scripts. OrthoMCL was run as described in the distribution manual using BLAST+ version 2.2.27 with an expectation cut off set at <10^−10^. Groups of proteins specific to particular pathogen sets were extracted using the grep function in unix.

### Delivery of heterologous proteins to host cells by *Xanthomonas* strain DAR61454

The Cya reporter construct pVSPnPro+AA1-100-AvrBs2∶Cya was obtained from Brian Staskawicz at the University of California, Berkeley, USA. This construct had the *AvrBs2* promoter, the first 300 nucleotides of *AvrBs2* coding region [48] and the *Cya* domain from *cyclosin* gene of *Bordetella pertussis* [26] cloned as an AvrBs2_T3SS_∶Cya translational fusion cassette in the broad host range vector pVSP61 [49]. As a control, a vector with the *AvrBs2* promoter and Cya but lacking the AvrBs2 leader sequence (AvrBs2_Prom_-Cya) was used. This construct was cloned by amplification of the AvrBs2 promoter and Cya coding sequence to create *Bam*HI-*Nco*I and *Nco*I-*Eco*RI fragments, respectively, for cloning via a triple fragment ligation into an *Eco*RI-*Bam*HI cut vector. These constructs were introduced into *X. translucens* strain DAR61454 (rifampicin marked) by electroporation. The recipient strain DAR61454 was grown at 33°C while making electro-competent cells and during initial selection of transformants to overcome interference from endogenous restriction modification system present in this strain.

Strain DAR61454 containing the *AvrBs2_T3SS_*∶*Cya* expression vector (AvrBs2_Prom_-AvrBs2_T3SS_∶Cya) or a control vector without the AvrBs2 leader sequence (AvrBs2_Prom_-Cya), was grown at 30°C for 36 hrs, cells harvested and resuspended in 10 mM MgCl_2_ to a OD of 0.4 and infiltrated into leaf blades of three-leaf stage wheat seedlings using needleless syringe. Leaf disc sampling, cAMP extractions and protein measurements were performed as described previously [27] with some modifications. The cAMP levels in the extracted samples were measured with a cAMP Enzyme Immunoassay kit (Cayman Chemical Company) according to manufacturer's instructions and expressed as pmole of cAMP per mg of total protein. Total protein was measured using the Quick Start Bradford Dye Reagent as per the manufacturer's instructions (BioRad).

## Supporting Information

Figure S1 Placement of *Xanthomonas translucens* isolate DAR61454 in the *Xanthomonas* genus using phylogenetic analysis of the *gyrB* gene nucleotide sequence. DAR61454 groups with other *X. translucens* pv. *translucens* isolates although the support for differentiation from *X. translucens* pv. *graminis* is weak. Phylogenetic analysis was carried out at www.phylogeny.fr as detailed in the methods. Numbers of branches represent branch support based on approximate likelihood ratio testing.
(EPS)

File S1 Contains the accessions for the groups of proteins specifically found in cereal infecting xanthomonads via the OrthoMCL analysis. Each row of the file has a unique group identifier followed by the individual protein identifiers for entries in that group. Where possible the GenBank locus identifier for that protein has been used. For locus identifiers that did not fit the naming convention required by OrthoMCL the locus identifier prefix was adjusted and can be found in Table 1.
(TXT)
